# Aflatoxins in Maize from Serbia and Croatia: Implications of Climate Change

**DOI:** 10.3390/foods12030548

**Published:** 2023-01-26

**Authors:** Jelka Pleadin, Jovana Kos, Bojana Radić, Ana Vulić, Nina Kudumija, Radmila Radović, Elizabet Janić Hajnal, Anamarija Mandić, Mislav Anić

**Affiliations:** 1 Laboratory for Analytical Chemistry, Croatian Veterinary Institute, Savska Cesta 143, 10000 Zagreb, Croatia; 2 Institute of Food Technology, University of Novi Sad, Bulevar Cara Lazara 1, 21000 Novi Sad, Serbia; 3 Croatian Meteorological and Hydrological Service, Ravnice 48, 10000 Zagreb, Croatia

**Keywords:** aflatoxins, maize, Serbia, Croatia, climate change

## Abstract

Aflatoxins (AFs) represent the most important mycotoxin group, whose presence in food and feed poses significant global health and economic issues. The occurrence of AFs in maize is a burning problem worldwide, mainly attributed to droughts. In recent years, Serbia and Croatia faced climate changes followed by a warming trend. Therefore, the main aim of this study was to estimate the influence of weather on AFs occurrence in maize from Serbia and Croatia in the 2018–2021 period. The results indicate that hot and dry weather witnessed in the year 2021 resulted in the highest prevalence of AFs in maize samples in both Serbia (84%) and Croatia (40%). In maize harvested in 2018–2020, AFs occurred in less than, or around, 10% of Serbian and 20% of Croatian samples. In order to conduct a comprehensive study on the implications of climate change for the occurrence of AFs in maize grown in these two countries, the results of available studies performed in the last thirteen years were searched for and discussed.

## 1. Introduction

Mycotoxins are secondary fungal metabolites that contaminate a variety of agricultural products worldwide, causing numerous negative health effects in humans and animals, as well as economic losses. Given their global occurrence, toxicity, and economic impact, one of the most important groups of mycotoxins are aflatoxins (AFs). As of now, around 20 AFs have been identified, the most frequently detected aflatoxin in contaminated agricultural samples thereby being aflatoxin B1 (AFB1). The other three most common naturally occurring AFs are aflatoxin B2 (AFB2), G1 (AFG1), and G2 (AFG2), all of them generally absent in the presence of AFB1. These four AFs are primarily produced by toxigenic strains of the *Aspergillus* (*A*.) fungi genus, mainly *A. flavus, A. parasiticus,* and *A. nomius*. Besides AFs of the B and G groups, AFs of the M group, particularly aflatoxin M1 (AFM1), are very important members of the AFs group as well. AFM1 is a derivative of AFB1 found in human and animal milk if the food or feed is contaminated with AFB1 [1,2,3,4]. 

AFs have the highest acute and chronic toxicity of all mycotoxins known so far and can have numerous negative effects on humans and animals. They may lead to the development of diseases (aflatoxicoses) with teratogenic, genotoxic, immunosuppressive, and mutagenic consequences. Furthermore, the International Agency for Research on Cancer (IARC) included AFB1, AFB2, AFG1, AFG2, and AFM1 into the Group 1 human carcinogenic compounds. Among AFs, AFB1 is the most frequent natural contaminant with most pronounced toxic and carcinogenic effects [5,6]. Potential health impacts of AFs vary considerably and span from acute effects to chronic outcomes, depending on several factors such as species, age, gender, the amount of ingested AFs, prior health status, etc. Based on the European Food Safety Authority (EFSA) panel report, among aflatoxin-related health outcomes, liver carcinogenicity and child health threats have been recognized as increasingly important [7,8]. It is generally believed that AFB1 has no threshold dose below which no tumour would develop, so that only zero level of exposure can vouch for no risk. In order to protect human and animal health, maximum limits (MLs) for AFs in foodstuffs and feedstuffs have been established by various governments. The most rigorous maximum allowable levels set out by the European Commission are for AFB1 and AFs (sum of AFB1, AFB2, AFG1, and AFG2). MLs for AFB1 and AFS in maize, intended for sorting or other physical treatment prior to human consumption or use as an ingredient in foodstuffs, are 5 and 10 μg/kg, respectively [9]. According to the European Commission, the AFB1 ML in maize intended for all feed materials is 20 μg/kg [10]. Croatia, as a European Union member state, applies the above regulations to the full. However, even though Serbia as a European Union candidate should harmonize its regulations with those of the European Union, differences in MLs stipulated for certain mycotoxins still exist. In the Serbian Regulation [11], MLs for AFB1 and AFs in maize intended for human consumption are in accordance with the European Union Regulation [9], but the AFB1 ML stipulated for maize used as feed is 30 μg/kg [12].

Aflatoxins-producing fungi of the *Aspergillus* genus are usually found in areas with warm climate and can contaminate agricultural products before or after harvesting. The degree of AFs food and feed contamination depends on the genetic factor as well as microclimate, including product moisture content, water activity (a_w_), relative humidity, temperature, pH value, and substrate composition, which can all go in favour of fungal growth and AFs production. Prevailing environmental conditions, that is to say, climatic factors that characterize different geographical areas and vary on an annual basis, are of special importance for the latter production. Contamination of agricultural products hurts the economy of the affected region, primarily that of the developing countries in which the fungal growth prevention strategy during harvest and crop storage is mostly inadequate [13,14]. Weather conditions in warm and humid subtropical and tropical zones are ideal for the colonization and dominance of *Aspergillus* species in different types of cereals, primarily maize, resulting in the production of AFs. Circumstances optimal for AFs production are the temperature of 33 °C and the a_w_ of 0.99, while the growth of aflatoxigenic fungi require the temperature of 35 °C and the a_w_ of 0.95 [15].

In recent years, AFs have more and more frequently occurred in European agricultural products as well. Registered climate changes are recognized as the most significant factors responsible for the increasing AFs presence across European countries [16,17,18,19,20,21,22]. The recently published EFSA report [2] highlighted the need for continuous monitoring of AFs occurrence due to climate change that may increase their prevalence. Therefore, the main aim of this study was to investigate the influence of weather on AFs occurrence in Serbian and Croatian maize in a four-year period (2018–2021). In order to allow for a comparative insight into the occurrence of AFs in maize grown in these two countries in relation to weather, the results of available studies conducted in the last 13 years were searched for and discussed.

## 2. Materials and Methods

### 2.1. Serbia

#### 2.1.1. Samples

In total, four hundred (*n* = 400) maize samples were collected from Northern Serbia (Bačka, Banat, and Srem), representing the main maize-growing regions in Serbia. Maize samples were collected in 2018–2021 with 100 per each study for the year targeting at the most common maize hybrids. Maize sampling was conducted by official controllers in accordance with the rules stipulated by the Serbian [23] and European Union Regulations [24]. Depending on the moisture content, maize samples were taken immediately after harvesting (moisture content 12–14%) or after drying in dryers (if moisture content after harvest > 14%). Dependent on that, maize samples were taken from farmers or dryers, before storage in the producer’s facilities or further distribution.

Each year, after the collection of maize, representative samples were prepared at the Institute of food technology in Novi Sad. Briefly, approximately 10 kg of aggregate samples were homogenized using a Nauta mixer (model 19387, Nauta patenten, Haarlem, The Netherlands), quartered, and ground to a 1 mm particle size using laboratory mill (KnifetecTM 1095 mill, Foss, Hoganas, Sweden). Obtained laboratory samples of 150–200 g were stored in zip lock bags at −18 °C until further processing. At the beginning of 2022, laboratory samples were taken from the freezer, again homogenized (Rotary laboratory mixer RRM Mini-II, Ludwigshafen, Germany) and quartered to get samples for analysis. In order to maintain the originality of the samples, and to avoid cross and secondary contamination, samples were stored in the freezer and each manipulation step (homogenization, grinding, quartering, packaging, and preparation) was done by qualified and experienced laboratory staff.

#### 2.1.2. Analysis

Maize samples were analysed using high-performance liquid chromatography-tandem mass spectrometry (LC-MS/MS). The following chemicals were used: acetonitrile of high-performance liquid chromatography (HPLC) grade (Fisher Scientific, Geel, Belgium); ultra-pure water (Adrona Crystal EX HPLCWater Purification system, Riga, Latvia); methanol (Carlo Erba, Val de Reuil, France), water (Fisher Scientific, Geel, Belgium), and formic acid (Fluka Analytical, Sigma Aldrich, Steinheim, Germany) of a LC-MS quality. Aflatoxin standards in the concentrations of 2.04 μg/mL for AFB1, 0.501 μg/mL for AFB2, 2.08 μg/mL for AFG1, and 0.504 μg/mL for AFG2, were purchased from Biopure (Romer Labs Division Holding GmbH, Tulln, Austria). Standard stock solutions were prepared by diluting aflatoxin standards in methanol/water (50:50, *v*/*v*). Correspondingly, matrix-matched standards were prepared by diluting an appropriate volume of an adequate standard stock solution in a blank sample extract and methanol/water (50:50, *v*/*v*), yielding the concentration levels of 0.050 to 30.0 µg/L for AFB1; 0.120 to 7.36 µg/L for AFB2; 0.102 to 30.6 µg/L for AFG1; and 0.247 to 7.41 µg/L for AFG2. Aflatoxin standards and stock solutions were stored in a freezer at −18 °C pending analysis.

Sample preparations were conducted in line with the method published by Hofmann and Scheibner [25]. Approximately 5 g of a ground sample was weighed in a 50 mL-polypropylene tube. The addition of 20 mL of acetonitrile/water (80/20, *v*/*v*) was followed by horizontal shaking at 5.8 Hz for 60 min and centrifugation at 39,240 m/s^2^ for 5 min at the room temperature. The supernatant was filtered through a 0.2 µm-PTFE disposable syringe filter. A 400 μL aliquot of the filtered supernatant was transferred into HPLC vials and diluted with 600 μL methanol/water (50/50, *v*/*v*) for LC-MS/MS analysis.

The detection and quantification were performed using a HPLC Vanquish Core system (ThermoFisher Scientific, Waltham, MA, USA) equipped with heated electrospray ionization (HESI) source and a TSQ Quantis Triple Quadrupole mass spectrometer (ThermoFisher Scientific, Waltham, MA, USA). The analysis utilized parameters outlined in the ThermoFisher Scientific (2021) with minor modifications. Chromatographic separation was performed at 40 °C on a ZORBAX Eclipse Plus C18-column, 100 × 2.1 mm i.d., 1.8 μm particle size (Agilent Technologies, Santa Clara, CA, USA). The mobile phases consisted of LC-MS grade water (mobile phase A) and methanol (mobile phase B), each supplemented with 0.1% LC-MS grade formic acid. The organic phase (mobile phase B) percent-share was modified throughout the analytical run according to the following specification: 0 min, 5%; 0.5 min, 5%; 7 min, 70%, 9 min, 100%; 12 min, 100%; 12.1 min, 5%; 15.0 min, 5%. The total instrument method run time was 15 min per sample. The column temperature was 40 °C, while the flow speed equal to 0.3 mL/min. The autosampler tray temperature was set at 20 °C with an injection volume of 10 µL. The mass spectrometer was operated in the selective reaction monitoring (SRM) mode with HESI in positive mode (3.5 kV). Nitrogen was used as a sheath, auxiliary, and sweep gas. Argon was used as a collision gas. The sheath gas was set at 30 Arb, the auxiliary gas at 6 Arb, the sweep gas at 1 Arb, while the collision induced dissociation (CID) gas pressure was adjusted to 1.5 mTorr. The ion transfer tube was set at 325 °C, with the vaporizer temperature of 350 °C. The cycle time was 0.5 s. Data were acquired and processed using the Thermo Scientific TraceFinder Software TSQ Quantis 3.2 Tune (Thermo Fisher Scientific, Waltham, MA, USA).

The LC-MS/MS method applied for AFs determination was validated in accordance with the European Regulation [26] and the Technical Report [27] of the European Committee for Standardization. The proposed method was validated for its limit of quantification (LOQ), linearity, trueness, recovery, repeatability, and reproducibility. The validation study was performed by analysing the quality control material (product code FCMA2-CCP30) provided by a world renowned accredited proficiency testing provider Food Analysis Performance Assessment Scheme (FAPAS) and spiked uncontaminated maize samples. The maize flour, serving as the quality control material, contained 3.50, 1.71, 1.78, 0.90, and 7.32 µg/kg of AFB1, AFB2, AFG1, AFG2, and AFs, respectively. Matrix effects were compensated by virtue of matrix-matched calibration (MMC) and calculated as signal suppression/enhancement (SSE), i.e., the MMC and solvent calibration (SC) curves slope ratio. For each aflatoxin, three characteristic product ions were monitored. First, the most abundant product ion was used for quantification, while the second and third ions were used as qualifiers for each aflatoxin: AFB1 (285.0, 241.0, and 269.0), AFB2 (259.0, 243.0, and 271.0), AFG1 (243.0, 200.0, and 215.0) and AFG1 (313.0, 189.0, and 285.0). The retention times for the investigated AFs were the following: AFG2, 8.89 min; AFG1, 9.20 min; AFB2, 9.55 min; and AFB1, 9.84 min.

The obtained validation parameters comply with the recommendation given under the Regulation 2006/401/EC [24] and the Technical Report [27]. Since the SSEs were higher than ±20%, all the toxins were quantified using MMC curves. The squared correlation coefficients (R^2^) were above 0.998 for all curves. LOQs for AFB1, AFB2, AFG1, and AFG2 were 0.5, 1.2, 1.0, and 2.5 µg/kg, respectively. Repeatability and reproducibility precision is expressed in the form of relative standard deviations, none of them exceeding 20%. Furthermore, both of the values, trueness and recovery, for all four AFs were in accordance with the stipulated performance criteria (i.e., between 83% and 114%). 

### 2.2. Croatia

#### 2.2.1. Samples

A total of 433 maize samples were obtained during 2018–2021 from different maize producers located in four Croatian regions (Central Croatia, *n* = 117; Eastern Croatia, *n* = 176; Northern Croatia, *n* = 110; and Western Croatia, *n* = 30). Whenever possible, during the four-year study period, the samples were taken from the same localities (production areas) each year dependent of the production capacities. They were obtained directly from farmers or medium size family enterprises (information on maize genus lacking) immediately after the process of maize drying in dryers (moisture content 12–14%) and before their storage in the capacities of the manufacturers or further distribution to domestic industries. Sampling and sample preparation were performed fully in line with the provisions of the Commission Regulation No. 401/2006 [24], stipulating the sampling methods to be exercised within the frame of monitoring of the mycotoxin levels in food. Aggregate maize samples consisted of three incremental samples weighing at least 1 kg. The prepared 500 g-laboratory test portions were ground into fine powder having a particle size of 1.0 mm using an analytical mill (Cylotec 1093, Tecator, Sweden) and stored at 4 °C prior to AFB1 analysis, which was performed within a maximum of 72 h.

#### 2.2.2. Analysis

The analyses of maize samples were carried out in the laboratory of the Croatian Veterinary Institute. Firstly, all samples were analysed using the ELISA method. In samples in which AFB1 content, determined using the ELISA method, exceeded 5 µg/kg (i.e., >ML for maize as food), further confirmatory analyses were performed using LC-MS/MS. The both applied methods, ELISA and LC-MS/MS are validated as also accredited in agreement with the ISO/IEC 17025 Standard [28]. For the purposes of the ELISA assay, maize grains were prepared using 5 g of the homogenized sample supplemented with 25 mL of methanol/water (70:30, *v*/*v*), and shaken vigorously head-over-head on a shaker for three minutes. The extract was then filtrated (Whatman, black ribbon) and 1 mL of the obtained filtrate was diluted with the appropriate volume of deionized water. The ELISA method was performed using a ChemWell 2910 auto-analyser (Awareness Technology, Inc., Palm City, FL, USA). In all maize samples, AFB1 content was determined using the ELISA method that made use of Ridascreen^®^ kits (Aflatoxin B1; Art. No. R1211) provided by R-Biopharm (Darmstadt, Germany); the procedure was carried out in full line with the instructions of the kit manufacturer. The obtained AFB1 content were calculated from a six-point calibration curve taking thereby the applied dilution factor into due account, and then corrected for the recovery value. The LOQ value of the applied ELISA method is 1.5 µg/kg. The implemented ELISA method was validated earlier in the research of Pleadin et al. [21].

For the purposes of the LC-MS/MS method, AFB1 standard was purchased from Sigma Aldrich (Saint Louis, MO, USA). AFB1 stock solution was prepared by dissolving 1 mg of the standard in 10 mL of acetonitrile (100 µg/mL). PuriTox Total Myco-MS solid phase clean-up columns were produced by R-Biopharm (Glasgow, Scotland). All chemicals for sample preparation and LC-MS/MS analyses were of a HPLC grade. Ultrapure water was supplied by the Merck system Direct-Q3 UV (Merck, Rahway, NJ, USA). Maize samples (2.5 g) were weighed into PTFE conical tubes followed by the addition of 10 mL of acetonitrile/water (80:20, *v*/*v*). Samples were vigorously shaken on a vortex shaker during 3 min, and then centrifuged during 10 min at the room temperature and 39,240 m/s^2^. Glacial acetic acid (20 µL) was added to 2 mL of the sample extract. After vortexing, 1.4 mL of an acidified sample extract was filtered through the PuriTox Total Myco-MS columns (R-Biopharm, Glasgow, Scotland). The filtrate (500 µL) was diluted with 1500 µL of acetic acid/water (1:99, *v*/*v*); the diluted filtrate was then supplemented with 100 µL of acetic acid/acetonitrile/water (1:20:79, *v*/*v*).

The instrumentation consisted of a HPLC (degasser, binary pump, auto-sampler, and column compartment, 1260 Infinity) and a mass spectrometer (QQQ 6410), all delivered by Agilent Technologies (Santa Clara, CA, USA). The mobile phase consisted of 0.1% formic acid and methanol (B). A gradient elution was employed as follows: 0–0.30 min 80% A, 0.30–4 min 60% A, 4–8 min 5% A, 8–10 min 5% A, with the flow rate of 0.5 mL/min and the column temperature of 40 °C. The injection volume was 15 µL, while the run time equalled to 15 min. The mass spectrometer with electrospray ionization (ESI) interface was operated in the Multiple Reaction Monitoring (MRM) mode. The ionization was performed in the positive ion mode (ESI +), with the source temperature set at 350 °C, the gas flow rate set at 9 L/min, the nebulizer set at 45 psi, and the capillary voltage set at 6000 V (+) and 3000 V (−). One precursor and two product ions were monitored, the protonated molecular ion of AFB1 at *m*/*z* = 313.0 being the precursor ion and ions at *m*/*z* = 241.0 and *m*/*z* = 285.0 being the product ions. 

The obtained AFB1 (retention time 12.68 min) content was calculated from a six-point calibration curve taking into account the applied sample dilution factor. Recovery was determined by spiking of the uncontaminated sample of maize flour at three different levels (5, 10 and 50 µg/kg) with the prepared standard AFB1 working solution (100 µg/L) adopted for in-house use (six replicates per concentration level). The mean recovery at levels 5, 10 and 50 µg/kg was 82.3, 95.4 and 97.9%, respectively. LOQ was calculated from the calibration curve in accordance with the guidance. Methods for the determination of the limit of detection and limit of quantitation of the analytical methods [29], and equalled 0.70 µg/kg. For the sake of the method quality control, the reference material (RM) in terms of cereal-based animal feed (Art. No. T04249QC, FAPAS, Sand Hutton, UK) with the assigned AFB1 value of 12.0 µg/kg (range, 6.7–17.2 µg/kg) was analysed with every sample batch. The trueness was determined on the same RM and expressed as recovery (94%). Notably, the laboratory is subject to proficiency testing once a year. 

### 2.3. Weather Analysis for Serbia and Croatia

Due to the fact that weather conditions, during the maize growing season, represent a factor with a strong influence on the presence or absence of AFs in maize, detailed analysis of weather condition parameters was conducted. The following parameters, that may have significant influence on the AFs occurrence, were considered: temperature (average air temperature, number of days with temperatures higher than 30 and 35 °C), precipitation (number of days with precipitation, the precipitation sum and deviation from that sum), and drought indicators (Palmer Drought Severity Index (PDSI), Standardized Precipitation Index (SPI-2), and Palmer Z Drought Index). The above data were recorded for 2018–2021 maize-growing seasons, i.e., from 1 April to 30 September. Deviations were determined by comparing these data to those recorded in a longer period (1981–2010). Data for Serbia and Croatia were provided by the Republic Hydrometeorological Service of Serbia [30] and Croatian Meteorological and Hydrological Service [31], respectively.

### 2.4. Moisture Determination

Moisture content was determined in accordance with International Standard Organization in all maize samples from Serbia [32] and Croatia [33].

### 2.5. Statistical Analysis

Microsoft Excel 2010 was used for statistical analysis of the data obtained in the validation study, as well as for the analysis of aflatoxins occurrence, and weather conditions parameters. For that purpose, the following functions were used: Average, percentage, median, maximum, minimum, standard deviation, and sum. The statistical analysis of data, on the occurrence of aflatoxins, was performed only on positive samples in which the determined content of AFs was higher than the LOQs of applied methods.

## 3. Results and Discussion

Natural occurrence of AFs was analysed in 400 Serbian and 433 Croatian maize samples collected over four years (2018–2021). The obtained results are interpreted in relation to the recorded weather data.

### 3.1. Serbia

#### 3.1.1. Aflatoxin Occurrence in 2018–2021 

The results presented in Table 1 reveal annual differences in natural occurrence of AFB1, AFB2, AFG1, AFG2, and AFs in maize samples collected in Serbia within the 2018–2021 timeframe. Besides, the mean moisture content for all maize samples per investigated year was shown in Table 1. 

Among the 100 maize samples analysed in each of the four study years, 8%, 11%, 5%, and 84% of maize samples were contaminated with AFs in 2018, 2019, 2020, and 2021, respectively. The highest aflatoxin content and the highest contamination frequency were recorded in 2021, while the proportion of aflatoxin-contaminated samples seen in other study years was approximately the same (around 10%). Each study year, the aflatoxin most commonly detected in the contaminated samples was AFB1. Only one 2018 sample was co-contaminated with AFG1 and AFG2, as well, while 2019 and 2020 samples were not co-contaminated at all. On the contrary, 2021 maize samples were contaminated with all four investigated AFs. AFB1, AFB2, AFG1, and AFG2 were detected in 84%, 26%, 20%, and 10% of 2021 maize samples, respectively. AFB1 was detected in the highest individual (0.5 to 246.3 µg/kg), mean (30.5 ± 41.0 µg/kg), and median (13.2 µg/kg) content. 

Even 61% of the examined 2021 maize samples were not suitable for human consumption, since the content of AFB1 and AFs were higher than the MLs of 5 and 10 µg/kg, respectively. Furthermore, due to the AFB1 content higher than 20 µg/kg, 34% of 2021 samples was unsuitable for animal consumption according to the European Regulation [10], and 27% according to the Serbian Regulation (aflatoxin content surpassing 30 µg/kg) [12]. In 2018–2020 maize samples, AFs were found in lower content. In 2% of 2018 and 3% of 2019 maize samples, AFB1 concentrations were higher than 5 µg/kg, making maize unsuitable for human consumption. The content of the other AFs determined in 2018, 2019, and 2020 maize samples were lower than MLs stipulated under both Serbian and European Union Regulations [9,10,11,12].

More and more often, the occurrence of AFs in Serbian maize comes as a response to stress caused by weather spells, especially weather extremes [16,17,18,19]. Therefore, the interpretation of differences in the obtained results called for the analysis of weather witnessed in the four study years.

#### 3.1.2. Weather Conditions in 2018–2021

The most important weather parameters descriptive of the maize-growing seasons (April–September 2018–2021) are summarized in Table 2. It can be noted that there is a difference in the number of days in which the temperature was higher than 30 and 35 °C, registered in the year 2021, compared to the remaining three study years. Furthermore, the 2021 maize-growing season was characterized with a considerably lower amount of precipitation in comparison to the other three study years.

Due to the fact that the weather descriptive of 2021 maize-growing season differed from that in other maize seasons analysed within this study frame, yielding the highest AFs contamination frequency, weather parameters pertaining to the summer months of 2021 were additionally analysed and shown in Table 3. 

As can be seen from Table 3, June, July, and August 2021, that is to say, the generative phase of maize, was characterized with hot and dry weather. Deviations in average air temperatures of 1.8 °C in June, 3.1 °C in July, and 0.7 °C in August, indicate that these three summer months of 2021 were warmer as compared to the summer months in a longer previous period (1981–2010). Higher air temperatures were followed by a lower amount of precipitation in June and August. Although a higher amount of precipitation was recorded in July 2021 as compared to the average value of 1981–2010, the precipitation was often local and came in form of rainstorms. Furthermore, during July, two approximately 15 days-lasting heat waves with temperatures of around 40 °C were recorded. As can be seen from Table 3, drought indicators (SPI-2, Z, and PDSI) show that June and August 2021 were both dry, as opposed to July during which the weather can be characterized as normal. Weather seen in September 2021 was also analysed for prolonged droughts capable of affecting 2021 maize-growing season. Just like in the previous months, during September 2021, a higher average air temperature and lower amount of precipitation was registered. The analysis indicates that September 2021 was characterized with extreme droughts judging by the Z indicator, while the SPI-2 and PDSI indicators labels these droughts as moderate.

### 3.2. Croatia

#### 3.2.1. Aflatoxin Occurrence in 2018–2021

The occurrence of AFB1 determined in Croatian regions during 2018–2021 as well as the mean moisture content of maize samples per investigated regions and years are shown in Table 4.

The results show the presence of AFB1 in 14% (2018), 16% (2019), 19% (2020), and 40% (2021) of maize samples. Five out of 433 samples (1%) collected in three Croatian regions contained this mycotoxin in levels higher than the ML of 20 µg/kg defined for maize in feed [10], while 22 samples (5%) harboured the mycotoxin in content higher than the ML of 5 µg/kg defined for maize in food [9]. The highest number of samples containing AFB1 in content higher than the ML was observed in the Eastern Croatia, which is the Croatian leader in grain production, farming, and milk production. The maximal AFB1 content detected in this region was 422.2 µg/kg, that is to say, roughly 20-fold higher than the ML stipulated for maize intended to be used as a feed component [10] and about 84-fold higher than the level of 5 μg/kg allowed in maize as food [9]. In the Central, Northern and Western regions, higher AFB1 levels were determined as well, but the number of samples that contained AFB1 in levels over the ML was lower. Additionally, many of the analysed samples harboured AFB1 in content slightly higher than, or around, the ELISA’s limit of quantification (1.5 µg/kg). A considerably higher level of contamination was seen in maize cultivated in 2021 in all regions, which can be attributed to the weather descriptive of the period important for maize cultivation. Namely, in 2021 the highest number of days in which the temperature rose above 35 °C and the lowest level of precipitation were registered in comparison with the three preceding years (2018–2020). It can be noted that the annual AFB1 incidence documented in the four-year study period can be linked to the weather.

#### 3.2.2. Weather Conditions in 2018–2021

The most important weather parameters registered in Croatia in maize-growing seasons (April to September 2018–2021) are summarized in Table 5. The data show that 2021 had a noticeably higher number of days in which the temperature exceeded 35 °C, while the number of days in which the temperature was over 30 °C was similar to that in 2019. In comparison with the preceding years (2018–2020), 2021 had a lower number of rainy days. Furthermore, the 2021 maize-growing season was the driest as compared to the remaining three study years. 

Due to the fact that, just like in Serbia, the highest AFB1 contamination frequency was established in 2021 in Croatia, as well, weather in the summer months of 2021 was additionally analysed and presented in Table 6. As was the case in Serbia, the maize generative phase in Croatia in 2021, also falling in June, July, and August, was characterized with hot and dry weather, too. As can be seen from Table 6, deviations in average air temperatures of 3.1 °C in June, 2.3 °C in July, and 0.2 °C in August indicate that the summer months of 2021 were warmer than those in 1981–2010. Higher air temperatures were followed by a lower amount of precipitation in June and August, while the amount of precipitation seen in July was slightly higher than the 1981–2010 average but still insufficient to mitigate the effects of drought. According to the SPI-2 and Z drought indicators, drought was recorded in July and September, while PDSI points towards drought during all analysed months in 2021. Prolonged drought conditions that continued during September were not suitable for maize, which requires a large amount of water during growing season. The analysis of 2021 meteorological data reveal that the weather was suitable for the synthesis of AFB1 in Croatian lowland, explaining the high AFB1 contamination frequency documented in that year. 

### 3.3. Comparative Study

Previous scientific studies published before 2010, indicate that AFs mainly contaminate agricultural products in tropical and subtropical regions (Africa, Australia, South and Southeast Asia), in which the local weather goes in favour of *Aspergillus* species colonisation and aflatoxin synthesis, while in Europe AFs occur less frequently [34,35,36,37]. However, in recent years, mainly due to the climate changes, AFs have more and more frequently contaminated various agricultural products in Europe [16,17,18,19,20,21,38,39,40]. Research shows that the interaction of climate changes, such as an increased carbon dioxide concentration, temperature rise, and extreme changes in length of dry and rainy periods, have a significant impact on fungal growth and the occurrence of AFs [41,42]. Therefore, with the aim of introducing a comparative insight into the implication of climate changes on the occurrence of AFs in maize from Serbia and Croatia, the results from available studies are investigated.

In the previous decade, Serbia and Croatia, as almost every European country located in central and southeast Europe, also faced climate changes followed by repeated weather extremes. In some years, climate changes mirrored in a warming trend characterised with an accelerated temperature increase, the absence of precipitation for several months, and droughts (2012, 2013, 2015, 2017, and 2021), while in other (2010, 2014 and 2019) abundant rainfalls that caused overflows and flooding were seen. In the previous decade, a warming trend that brings increased air temperatures was registered almost every year. It is well known that frequent heat disturbances may affect human health, agricultural production, food, water supplies, and many other areas vital for economic and social well-being. Drastic agricultural consequences, and economic losses incurred due to, the prolonged droughts and record summer temperatures, were already documented in both Serbia and Croatia. During the last decade, climate changes registered in both countries already manifested their influence through significant challenges to, and difficulties in, agriculture, food production, and food safety. In the agricultural sector, climate changes followed by increasing temperatures and reduced water availability have the greatest impact on, and the greatest consequences for, crop production. In light of the substantial climate change, the production, yields, quality, and safety of maize in Serbia and Croatia, are vastly challenged. At the same time, weather extremes are directly responsible for the decrease in maize yields and its increased contamination with certain mycotoxins, such as AFs [16,17,18,19,20,21,43,44,45,46,47]. The results of the studies conducted in the last decade indicate that AFs frequently occur in certain parts of Europe. In all of these studies, the authors highlighted climate changes, warming trend and prolonged droughts as the main reasons behind the increased AFs prevalence and contamination frequency. Among agricultural products of Serbia and Croatia, the greatest influence of climate change on the prevalence of AFs was noticed in maize [16,17,18,19,20,21,22].

**Figure 1:**
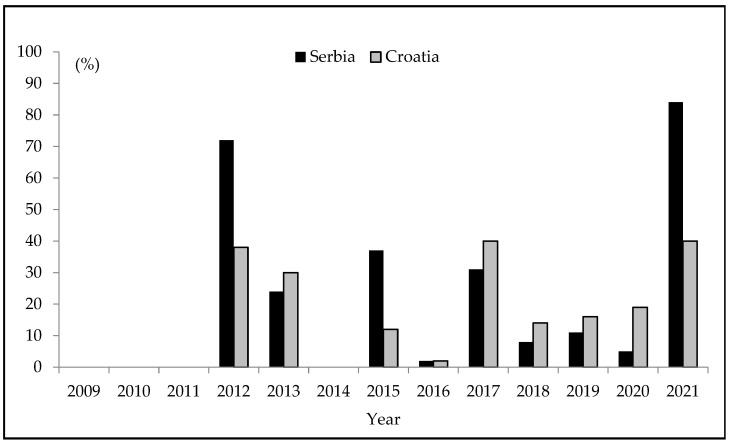
An overview of the percentage of contaminated maize samples with AFs in Serbia and AFB1 in Croatia in 2009–2021.

Figure 1 represents an overview of the occurrence of AFs and AFB1 in maize collected in Serbia and Croatia in 2009–2021, respectively. The occurrence data are shown as a percentage of contaminated samples in which the determined content was greater than LOQs of the applied methods. In Figure 1, data on the following Serbian maize-growing seasons were summarized: 2009–2011 [16]; 2012–2015, [17]; and 2016–2017 [19]. As for Croatia, the 2009–2013 maize-growing seasons are embraced [20,21]. Data earlier unpublished and data obtained in this study are included as well. 

As can be seen from Figure 1, AFs were not detected in Serbia neither AFB1 in Croatia in four (2009–2011, 2014) out of thirteen years under this review. In Serbia in three years (2016, 2018, and 2020), and in Croatia in two years (2016 and 2018), presence of investigated aflatoxins was registered in less than or around 10% of maize samples. Contrary to this, considerably higher levels of aflatoxin contamination of 72%, 24%, 37%, 31%, and 84% were detected in maize samples from Serbia harvested in 2012, 2013, 2015, 2017, and 2021, respectively. Based on the obtained results, it can be assumed that Serbia becomes susceptible to AFs presence-evoked problems, since AFs were detected in Serbian maize in nine out of thirteen years embraced by this review, in five out of these nine years even in high prevalence rates ranging from 24% to 84%. These substantial deviations from AFs occurrence patterns, seen in maize samples collected in different years, can be explained by the fact that in the thirteen year review period Serbia had experienced climate changes. As for Croatia, it can be concluded that the percentage of positive samples uncovered in the referent period was lower than in Serbia, but the occurrence of AFB1 in the last thirteen years was still significant, with the maximum of 40% of positives in 2021 and 38% of positives in 2012. While the occurrence of AFB1 evidenced in 2012 was slightly lower compared to 2021 and even more so to other years under this review, it is important to emphasise that in 2012 this mycotoxin was present in maize in extreme content [21], never recorded in Croatia either before or later.

The analysis of Palmer Drought Severity Index descriptive of the summer months of the years, in which the prevalence of AFs in maize was higher, is shown in Table 7 for Serbia and in Table 8 for Croatia. 

From Figure 1 and Table 7, it can be noted that a higher prevalence of AFs in Serbia was detected in years characterised with more pronounced droughts. In June, July, and August 2012, extreme droughts were registered, downsized to moderate in September. Daily temperatures during these summer months were very often close to 40 °C; at some locations, the temperature deviated from the average by even 6 °C. Heat waves and a considerably lower level of precipitation in comparison with 1981–2010, mirrored in a low moisture reserve. The amount of precipitation increased in late July, but unfortunately more than half of the maize crops were already damaged. Lević et al. [48] reported that extremely stressful agrometeorological conditions recorded in the summer of 2012 went in favour of *A. flavus,* so that its representation rose to 95%. Furthermore, drought enhances the appearance of various pests, so that all these factors combined greatly influenced aflatoxin synthesis. As can be seen from the Table 7, in other years in which maize samples were also contaminated with AFs (2013, 2015, 2017, and 2021), extreme droughts were not recorded (2013 and 2015), or rarely appeared in certain months (2017 and 2021). In 2013, the weather was mainly described as normal, while in 2015 and 2017 dominated moderate and severe drought episodes. Based on these facts, it can be argued that differences in aflatoxin contamination levels are attributable to differences in drought occurrence frequencies. In these five years, the detected AFs content varied in the following descending order: 2012 > 2021 > 2017 > 2015 > 2013. In years with no drought periods (2009, 2010, 2011, 2014), AFs were not detected, or detected in less than 5% (2016) [16,17,18,19].

Unfortunately, in each year in which AFs were detected in maize, AFM1 contaminated Serbian milk and dairy products. Dietary exposure to AFM1 in Serbia, coming as a result of milk consumption, was elaborated within the frame of several studies. Due to the high contamination frequency and high AFM1 concentrations in milk, certain age groups, especially children, were at high contamination and health risk. Due to the variability of AFB1 content in maize and consequently of AFM1 in milk across the sampling years, inter-annual exposure level differences were huge as well [16,17,18,19,49,50,51].

Despite of the high aflatoxin contamination frequency, documented in five out of the last thirteen years, in Serbia these toxins received the greatest public attention in 2012 and 2013. At the time, the public was informed about AFs contamination of maize and milk through different media. Public concern grew rapidly due to a lot of easily accessible contradictory information offered by the media. In early 2013, Serbia faced the “aflatoxins crisis” followed by the protest of agricultural workers, the replacement of the Minister of Agriculture, several amendments to the Regulation governing the maximum level of AFM1 and AFB1, confusion among consumers, and decrease in purchase of milk and dairy products. The “aflatoxins crisis” was ultimately responsible for a significant economic loss (of about hundred million dollars). The results obtained in this study, as well as the results of our previous study, indicate that ten years after the “aflatoxins crisis” Serbia still faces aflatoxin-related problems [16,17,18,19,51,52,53].

In recent years, the presence of AFs and human exposure to these toxins raise a great concern in Serbia, since milk and maize are among the main foodstuffs, especially for children. Unfortunately, climate change predictions for this part of Europe indicate that warming trend favourable to *Aspergillus* species and aflatoxin synthesis will be continued in the future. Therefore, the agricultural sector in Serbia is facing a great challenge of rapidly growing food and feed contamination, which requires reinforced control strategies based on continuous monitoring, increasing investments, multidisciplinary approach, and education of all food chain participants, so as to minimize the presence of AFs in maize and consequently in the entire food and feed chain [52,53,54,55,56].

As for Croatia, previously registered weather changes characterized with heating trend, also manifested themselves in aflatoxin maize contamination. The Palmer Drought Severity Index (Table 8) values show that, in comparison with other years of the last decade, the weather in 2012 was extremely dry during all four months important for maize cultivation, resulting in the highest level of AFB1 maize contamination ever recorded in Croatia [21]. According to the PDSI, the weather in the same period of the year 2013 was normal, that in 2015 moderately to slightly wet, whereas in 2017 the weather in June and July was normal and slightly dry in August.

Given that elevated aflatoxin content are usually associated with humidity and temperature as the factors critical for fungi formation and thus AFs production [20,56], the results obtained by this study can be attributed to these factors, as well. High levels of AFB1 maize contamination seen in Croatia during 2012 and 2013 were mainly associated with weather, given that the Croatian Meteorological and Hydrological Service recorded the summer of 2012 as extremely warm and dry. Droughts and high temperatures seen during maize growth and harvest encourage the growth of *Aspergillus* species, with AFB1 production in the optimal temperature range of 25 to 42 °C [5]. Exactly the same weather, recorded in 2012 in the Republic of Croatia, caused the contamination of maize and cattle feed mixtures with AFB1 and, consequently, AFM1 contamination of milk coming from dairy cattle farms [20,21,57]. Bilandžić et al. [58] reported that extreme droughts witnessed in 2016 to 2022 contributed to the development of toxigenic fungi and the increased frequency of AFM1 milk contamination in Croatia. 

Besides the influence on fungal growth and aflatoxin synthesis, recent climate changes are also recognized as the main factors responsible for the reduction of maize yields in both Serbia and Croatia. In Serbia, in years 2012, 2013, 2015, 2017, and 2021 in which AFs were detected frequently, the maize yield dropped below the ten-year average of around 6.2 Mt/ha. The historical maize yield minimum (of 3 Mt/ha) was registered in the year 2012, i.e., in the same year in which the highest occurrence of AFs was observed, while in years in which the weather was not dry maize yields very often exceeded 8 Mt/ha. The reduction of maize yields directly downsizes the amount of the exported maize. On average, Serbia exports 2.1 Mt of maize annually, varying from 0.6 Mt in 2012 to 3.1 Mt in 2019 and 2020 [59]. The influence of weather changes on maize yields was also noticed in Croatia in recent years. Maize yields varied from 4.3 Mt/ha in the year 2012 to 9.0 Mt/ha in 2019 [60]. It can be assumed that in both Serbia and Croatia recent global warming and more frequent droughts cause significant damage to maize production. A decrease in maize yields should be considered as an issue of the outmost significance, since in these two countries maize represents one of the most important agricultural items. About 40% of the total planted crop area in Serbia is populated by maize, mostly used for feed production (80%), while the remaining amount is intended for human consumption and food industry. With the ten-year average of 2.1 million tons intended for export, Serbia is ranked among the leading maize exporters both on the European and the global scale. However, in Serbia maize is largely grown on rainfed fields, since only 5–9% of the maize-growing land is irrigated. Maize is also one of the major field crops grown on arable lands of Croatia. Unfortunately, as in Serbia, Croatian maize is also mainly grown on an unirrigated land. Some authors claim that in Croatia weather characteristics, especially rainfall and temperature regimes represent the factor most responsible for maize yield oscillations over years. It is well known that maize needs a large amount of water due to its large vegetative mass, high yields, and long growing season; therefore, attention should be focused on irrigation improvements, especially in view of recent heat waves and droughts emerging during maize-growing seasons. Recent global warming and more frequent droughts have already caused a considerably damage to maize production in Serbia and Croatia [43,59,60]. 

Besides Serbia and Croatia, other European countries have also recently faced climate change that significantly affects their agricultures. A lot of reports have confirmed the heating trend in Europe [61,62,63,64,65,66,67]. Battilani et al. [61] reported that future climate changes are expected to have an impact on the presence of AFB1 in European maize. The authors used a modelling approach to predict aflatoxin maize contamination at increasing temperatures and showed that a +2 °C climate change scenario would increase the probability of aflatoxin contamination from low to medium in European countries in which maize cultivation is common. Further, the Annual Report of the European State of Climate [68], published by the Copernicus Climate Change Service in 2021, indicates that between 1991 and 2021 temperatures in Europe rose by 0.5 °C per decade on the average, as compared to the global average of 0.2 °C. This report points out that warming trend will be continued and followed by an exceptional heat, extreme drought, wildfires, floods, and other climate breakdown outcomes that will affect society, economies, and ecosystems. Furthermore, according to the report of the World Meteorological Organization, in the last 30 years temperatures in Europe have increased by more than twice the global average. In the year 2021, in which hot and dry weather influenced the frequency of AFs presence in Serbian and Croatian maize, almost the whole of Europe also faced a record number of extreme heat stress days, while the area much larger than usually experienced a strong to moderate heat stress. The area affected by a strong heat stress was at least twice the average [69].

## 4. Conclusions

The findings of this study reveal an evident implication of climate change on AFs occurrence in maize, the main field crop of both Serbia and Croatia. In years, characterized as extreme wet (2014) and moderate conditions (2009, 2010, and 2011) aflatoxins were not contaminated maize from these two countries. However, the results obtained in this as well as in the comparative study indicate that in years in which the weather conditions were characterized by high temperatures, lack of precipitation and pronounced drought conditions, a more frequent occurrence of aflatoxins was observed. The following contamination frequency of aflatoxins was determined in Serbia: between 2 and 8% in 2016, 2018, 2019, and 2020 years; between 24 and 37% in 2013, 2015, and 2017; 72% in 2012 and 84% in 2021; and in Croatia 2% in 2016, between 12 and 19% in 2015, 2018, 2019, and 2020, and between 30 and 40% in 2012, 2013, and 2020 years. Based on this fact it could be noted that in the previous decade, AFs occurred very frequently in maize coming from the investigated regions. Therefore, for these two countries AFs have become a burning concern due to their increased prevalence. An increased AFs contamination of maize represents a global concern, which goes beyond a problem of local importance limited only to Serbia and Croatia, due to its significant role in the food and feed supply chain out of their borders. Since maize is a leading crop in Serbia and Croatia, grown predominantly on an unirrigated land, along with looking for climate change adaptation options, more frequent cultivation of drought-tolerant maize hybrids has a priority. The number of dry years, already increased in the recent decades due to global warming, will continue to rise and is a cause for concern. Climate change—introduced challenges require adaptation strategies that should encompass, in the first place, risk management plans founded on preventive and planned adaptation and innovation, including different changes in production systems, so as to maintain long-term productivity. To the best of the authors’ knowledge, this study represents the first report from Serbia and Croatia that provides a comparative insight into the occurrence of AFs in maize samples collected over a period of 13 years.

## Figures and Tables

**Table 1:** The occurrence of aflatoxins in maize samples collected in Serbia in 2018–2021.

Year	Aflatoxin	N ^1^ (%)	Min–Max ^2^	Mean ± Std ^3^	Median ^4^	Moisture ^5^
2018	AFB1 AFB2 AFG1 AFG2 AFs	8 (8) nd ^6^ 1 (1) 1 (1) 8 (8)	0.8–8.3 nd 1.7 3.1 8 (8)	3.6 ± 2.3 nd 1.7 3.1 8.1	3.3 nd 1.7 3.1 8.1	13.4
2019	AFB1 AFB2 AFG1 AFG2 AFs	11 (11) nd nd nd 11 (11)	0.6–10.9 nd nd nd 0.6–10.9	3.0 ± 2.5 nd nd nd 3.0 ± 2.5	1.6 nd nd nd 1.6	12.9
						
2020	AFB1 AFB2 AFG1 AFG2 AFs	5 (5) nd nd nd 5 (5)	1.1–3.0 nd nd nd 1.1–3.0	2.1 ± 0.7 nd nd nd 2.1 ± 0.7	2.2 nd nd nd 2.2	13.2
						
2021	AFB1 AFB2 AFG1 AFG2 AFs	84 (84) 26 (26) 20 (20) 10 (10) 84 (84)	0.5–246.3 1.8–13.9 1.2–173.9 2.7–30.7 0.5–246.3	30.5 ± 41.0 3.9 ± 3.1 26.0 ± 53.2 7.7 ± 9.5 38.8 ± 48.3	13.2 2.9 4.0 3.1 16.8	12.4

^1^ N (%): number (percentage) of contaminated samples; ^2^ Min-Max: minimum and maximum content (µg/kg); ^3^ Mean ± Std: mean content (µg/kg) ± standard deviation (µg/kg); ^4^ Median: median content (µg/kg); ^5^ Moisture (%) mean moisture content; ^6^ nd: not detected i.e., below the limit of quantification (LOQ).

**Table 2:** Weather parameters in Serbia registered in April–September of 2018–2021.

Year	N ^1^ T ^2^ > 30 °C	N T > 35 °C	N Precipitation	∑^3^ P ^4^ (mm)	∑ P (%)
2018	42	0	54	382	106
2019	36	1	54	424	118
2020	37	1	44	332	91
2021	50	13	44	296	81

^1^ N: numbers of days; ^2^ T: air temperature; ^3^ ∑ P (mm): sum of precipitation during period April–September; ^4^ ∑ P (%): ratio between sum of precipitation and long-term average (1981–2010) during period April–September.

**Table 3:** Weather parameters and drought indicators registered in Serbia in June–September 2021.

Month	T ^1^ (°C)		∑ P ^4^ (mm)		Drought Indicators					
	ΔTaver ^2^	Tmax ^3^	2021	1981–2010	SPI-2 ^5^		Z ^6^		PDSI ^7^	
June	1.8	38.1	49	83	−1.0	MoD ^8^	−3.9	ED ^10^	−2.2	MoD
July	3.1	37.3	99	63	−0.1	N ^9^	0.5	N	−1.6	N
August	0.7	37.2	44	56	0.5	N	−1.1	MoD	−2.0	MoD
September	1.4	33.2	16	52	−1.2	MoD	−2.8	ED	−2.8	MoD

^1^ T: air temperature; ^2^ ΔTaver: deviation from the average air temperature; ^3^ Tmax: maximum temperature; ^4^ ∑ P (mm): sum of precipitation; ^5^ SPI-2: Standardized Precipitation Index for 60 days; ^6^ Z: Palmer Z Drought Index; ^7^ PDSI: Palmer Drought Severity Index; ^8^ MoD: moderate drought; ^9^ N: normal; ^10^ ED: extreme drought.

**Table 4:** Aflatoxin B1 occurrence during 2018–2021 in four Croatian regions.

Croatian Region	Parameter	Year			
		2018	2019	2020	2021
Central	N ^1^ total	30	28	22	37
	N (%) of positives	4 (13)	3 (11)	3 (14)	14 (38)
	Min–Max ^2^	1.6–75.1	1.5–26.9	1.5–2.1	1.6–1.7
	Mean ± Std ^3^	6.9 ± 19.6	3.5 ± 6.8	1.6 ± 0.6	1.6 ± 0.2
	Median ^4^ Moisture ^6^ (%)	1.7 11.9	1.6 12.0	1.7 13.0	1.7 11.8
Northern	N total	29	27	29	25
	N (%) of positives	3 (10)	4 (15)	5 (17)	10 (40)
	Min–Max	1.7–25.4	1.5–2.7	1.6–2.1	2.2–23.2
	Mean ± Std	3.4 ± 5.8	1.7 ± 0.5	1.7 ± 0.5	9.3 ± 12.0
	Median Moisture (%)	2.0 12.1	1.7 12.1	1.7 12.0	2.5 11.7
Eastern	N total	45	48	41	42
	N (%) of positives	7 (16)	9 (19)	12 (29)	18 (43)
	Min–Max	1.7–31.2	1.5–13.3	1.5–3.2	1.8–422.2
	Mean ± Std	3.5 ± 6.8	2.3 ± 3.1	1.6 ± 0.6	54.6 ± 133.3
	Median Moisture (%)	2.0 13.0	1.7 12.4	1.6 12.1	2.4 11.9
Western	N total	6	6	11	7
	N (%) of positives	1 (17)	1 (17)	0 (0)	2 (29)
	Min–Max	1.6–68.2	1.5–1.7	^5^ nd	1.5–2.6
	Mean ± Std	40.4 ± 35.1	1.6 ± 0.5	2.0 ± 0.9	2.0 ± 1.1
	Median Moisture (%)	52.0 11.9	1.7 13.6	1.9 13.8	2.012.5
All regions (total)	N total	110	109	103	111
	N (%) of positives	15 (14)	17 (16)	20 (19)	44 (40)
	Min–Max	1.6–75.1	1.5–26.9	1.5–3.3	1.5–422.2
	Mean ± Std	6.2 ± 14.9	2.5 ± 4.3	1.6 ± 0.6	34.1 ± 103.2
	Median Moisture (%)	2.0 12.2	1.7 12.5	1.6 12.7	2.3 12.0

^1^ N: number; ^2^ Min-Max: minimum and maximum content (µg/kg); ^3^ Mean ± Std: mean content (µg/kg) ± standard deviation (µg/kg); ^4^ Median: median content (µg/kg); ^5^ nd: not detected i.e., below the limit of quantification (LOQ); ^6^ Moisture (%) mean moisture content. Results were obtained using the ELISA method, whereas content higher than 5 µg/kg were confirmed by LC-MS/MS method; in that case, the results of the LC-MS/MS method are presented.

**Table 5:** Weather parameters in Croatia in April–September 2018–2021.

Year	N ^1^ T ^2^ > 30 °C	N T > 35 °C	N Precipitation	∑ ^3^ P ^4^ (mm)	∑ P (%)
2018	35	0	45	442	93
2019	43	1	53	615	129
2020	31	1	47	462	96
2021	42	8	42	379	80

^1^ N: numbers of days; ^2^ T: air temperature; ^3^ ∑ P (mm): sum of precipitation during period April–September; ^4^ ∑ P (%): ratio between sum of precipitation and long-term average (1981–2010) during period April-September.

**Table 6:** Weather parameters and drought indicators in Croatia for June–August in 2021.

Month	T ^1^ (°C)		∑ P ^4^ (mm)		Drought Indicators					
	ΔTaver ^2^	Tmax ^3^	2021	1981–2010	SPI-2 ^5^		Z ^6^		PDSI ^7^	
June	3.1	36.3	17	97	−0.8	N ^8^	1.5	SW ^10^	−1.8	MiD ^13^
July	2.3	36.5	88	73	−1.3	SD ^9^	3.5	ED ^11^	−1.7	MiD
August	0.2	36.4	62	80	0.1	N	0.1	N	−1.9	MiD
September	0.7	32.1	40	80	−0.9	N	1.1	MoD ^12^	−2.2	MoD

^1^ T: air temperature; ^2^ ΔTaver: deviation from the average air temperature; ^3^ Tmax: maximum temperature; ^4^ ∑ P (mm): sum of precipitation; ^5^ SPI-2: Standardized Precipitation Index for 60 days; ^6^ Z: Palmer Z Drought Index; ^7^ PDSI: Palmer Drought Severity Index; ^8^ N: normal; ^9^ SD: severe drought; ^10^ SW: slightly wet; ^11^ ED: extreme drought; ^12^ MoD: moderate drought; ^13^ MiD: mild drought.

**Table 7:** Palmer Drought Severity Index established in Serbia for June to September 2012, 2013, 2015, and 2017.

Month	Palmer Drought Severity Index							
	Year							
	2012		2013		2015		2017	
June	−4.0	ED ^1^	0.7	N ^2^	−0.1	N	−3.0	SD ^4^
July	−4.2	ED	−0.5	N	−2.8	MoD	−3.9	SD
August	−4.5	ED	−2.1	MoD ^3^	−2.4	MoD	−4.0	ED
September	−2.0	MoD	−0.9	N	−2.0	MoD	−3.2	SD

^1^ ED: extreme drought; ^2^ N: normal; ^3^ MoD: moderate drought; ^4^ SD: severe drought.

**Table 8:** Palmer Drought Severity Index descriptive of June to September 2012, 2013, 2015, and 2017 in Croatia.

Month	Palmer Drought Severity Index							
	Year							
	2012		2013		2015		2017	
June	−5.1	ED ^1^	−0.2	N ^2^	2.2	MW ^3^	−0.5	N
July	−5.5	ED	−0.5	N	1.8	SW ^4^	−0.7	N
August	−6.1	ED	−0.7	N	1.6	SW	−1.2	MiD ^5^
September	−5.5	ED	−0.5	N	1.5	SW	0.2	N

^1^ ED: extreme drought; ^2^ N: normal; ^3^ MW: moderately wet; ^4^ SW: slightly wet; ^5^ MiD: mild drought.

## Data Availability

Data is contained within the article.

## References

[1] FAO/WHO, Food and Agriculture Organization of the United Nations/World Health Organization (2018). Aflatoxins. Safety evaluation of certain contaminants in food: Prepared by the eighty-third meeting of the Joint FAO/WHO Expert Committee on Food Additives (JECFA). WHO Food Addit. Ser..

[2] European Food Safety Authority (2020). EFSA CONTAM Panel, 2020. Scientific opinion—Risk assessment of aflatoxins in food. EFSA J..

[3] Abdulrauf L.B., Abdulrauf L.B. (2021). Aflatoxin Occurrences and Food Safety. Aflatoxins: Occurrence, Detoxification, Determination and Health Risks.

[4] Frisvad J.C., Hubka V., Ezekiel C.N., Hong S.B., Nováková A., Chen A.J., Mahakarnchanakul W., Samson R.A., Houbraken J. (2019). Taxonomy of Aspergillus section Flavi and their production of aflatoxins, ochratoxins and other mycotoxins. Stud. Mycol..

[5] International Agency for Research on Cancer (1993). Some naturally occurring substances: Food items and constituents, heterocyclic aromatic amines and mycotoxins. IARC Monograph on The Evaluation of Carcinogenic Risks to Humans, Vol. 56.

[6] International Agency for Research on Cancer (2012). Chemical agents and related occupations, a review of human carcinogens. IARC Monograph on the Evaluation of Carcinogenic Risk to Humans, Vol. 100F.

[7] European Food Safety Authority (2004). Opinion of the Scientific Panel on Contaminants in the food chain on a request from the commission related to aflatoxin B1 as undesirable substance in animal feed. Request No EFSA-Q-2003-035. EFSA J..

[8] European Food Safety Authority (2020). Climate change as a driver of emerging risks for food and feed safety, plant, animal health and nutritional quality. EFSA Support. Publ..

[9] European Commission (2006). European Commission Regulation No 1881/2006 of 19 December 2006 setting maximum levels for certain contaminants in foodstuffs. Off. J. Eur. Union.

[10] European Commission (2003). Commission Directive 2003/100/EC of 31 October 2003 amending Annex I to Directive 2002/32/EC of the European Parliament and of the Council on undesirable substances in animal feed. Off. J. Eur. Union.

[11] Serbian Regulation (2011). Maximum allowed contents of contaminants in food and feed. Off. Bull. Repub. Serb..

[12] Serbian Regulation (2017). Quality of animal feed. Off. Bull. Repub. Serb..

[13] Asefa D.T., Kure C.F., Gjerde R.O., Langsrud S., Omer M.K., Nesbakken T., Skaar I. (2011). A HACCP plan for mycotoxigenic hazards associated with dry-cured meat production processes. Food Control.

[14] Binder E.M., Tan L.M., Chin L.J., Handl J., Richard J. (2007). Worldwide occurrence of mycotoxins in commodities, feeds and feed ingredients. Anim. Feed Sci. Technol..

[15] Sanchis V., Magan N., Magan N., Olsen M. (2004). Environmental conditions affecting mycotoxins. Mycotoxins in Food.

[16] Kos J., Mastilović J., Hajnal E.J., Šarić B. (2013). Natural occurrence of aflatoxins in maize harvested in Serbia during 2009–2012. Food Control.

[17] Kos J., Janić Hajnal E., Šarić B., Jovanov P., Mandić A., Đuragić O., Kokić B. (2018). Aflatoxins in maize harvested in the Republic of Serbia over the period 2012–2016. Food Addit. Contam. Part B.

[18] Kos J., Hajnal E.J., Malachová A., Steiner D., Stranska M., Krska R., Poschmaier B., Sulyok M. (2020). Mycotoxins in maize harvested in Republic of Serbia in the period 2012–2015. Part 1: Regulated mycotoxins and its derivatives. Food Chem..

[19] Kos J., Janić Hajnal E., Radić B., Pezo L., Malachová A., Krska R., Sulyok M. (2022). Two years study of Aspergillus metabolites prevalence in maize from the Republic of Serbia. J. Food Process. Preserv..

[20] Pleadin J., Vulić A., Perši N., Škrivanko M., Capek B., Cvetnić Ž. (2014). Aflatoxin B1 occurrence in maize sampled from Croatian farms and feed factories during 2013. Food Control.

[21] Pleadin J., Vulić A., Perši N., Škrivanko M., Capek B., Cvetnić Ž. (2015). Annual and regional variations of aflatoxin B1 levels seen in grains and feed coming from Croatian dairy farms over a 5-year period. Food Control.

[22] Tóth B., Kótai É., Varga M., Pálfi X., Baranyi N., Szigeti G., Kocsubé S., Varga J. (2013). Climate change and mycotoxin contamination in Central Europe: An overview of recent findings. J. Agric. Rural. Dev..

[23] Serbian Regulation (1988). Methods for physical and chemical analysis for quality control of cereals, mills and bakery products, pasta and fast frozen dough. Off. Bull. SFRJ.

[24] European Regulation (2006). No 2006/401/EC of 23 February 2006 laying down the methods of sampling and analysis for the official control of the levels of mycotoxins in foodstuffs. Off. J. Eur. Union.

[25] Hofmann S., Scheibner O. (2021). Quantification of 48 myco- and phytoxins in Cereal Using Liquid Chromotography-Triple Quadrupole Mass Spectrometry. ThermoFisher Scientific, Application Note 65969. https://assets.thermofisher.com/TFS-Assets/CMD/Application-Notes/an-65969-mycotoxin-phytotoxins-cereal-tsq-quantis-an65969-en.pdf.

[26] European Commission (2002). Commission Regulation 2002/657/EC of 14 August 2002 implementing council directive 96/23/EC concerning the performance of analytical methods and the interpretation of results. Off. J. Eur. Communities.

[27] (2012). Food Analysis-Performance Criteria for Single Laboratory Validated Methods of Analysis for the Determination of Mycotoxins.

[28] (2017). General Requirements for the Competence of Testing and Calibration Laboratories.

[29] Shrivastava A., Gupta V.B. (2011). Methods for the determination of limit of detection and limit of quantitation of the analytical methods. Chron. Young Sci..

[30] Republic Hydrometeorological Service of Serbia. http://www.hidmet.gov.rs/ciril/meteorologija/agro.php.

[31] Croatian Meteorological and Hydrological Service. https://meteo.hr/index_en.php.

[32] (2012). International Standard Organisation (ISO): Maize—Determination of Moisture Content (on Milled Grains and on Whole Grains).

[33] (1980). International Standard Organisation (ISO): Maize—Determination of Moisture Content (on Milled Grains and on Whole Grains).

[34] Pitt I.I., Hocking A.D., Barug D., van Egmond H.P., Lopez-Garcia R., van Ossenbruggen T., Visconti A. (2004). Current mycotoxin issues in Australia and Southeast Asia. Meeting the Mycotoxin Menace.

[35] Turner P.C., Sylla A., Gong Y.Y., Diallo M.S., Sutcliffe A.E., Hall A.J., Wild C.P. (2005). Reduction in exposure to carcinogenic aflatoxins by postharvest intervention measures in West Africa: A community-based intervention study. Lancet.

[36] Shephard G.S. (2008). Risk assessment of aflatoxins in food in Africa. Food Addit. Contam..

[37] Karami-Osboo R., Mirabolfathy M., Kamran R., Shetab-Boushehri M. (2012). Aflatoxin B1 in maize harvested over 3 year in Iran. Food Control.

[38] Valencia-Quintana R., Milić M., Jakšić D., Šegvić Klarić M., Tenorio-Arvide M.G., Pérez-Flores G.A., Stefano B., Sánchez-Alarcón J. (2020). Environment changes, aflatoxins, and health issues: A review. Int. J. Environ. Res. Public Health..

[39] Moretti A., Pascale M., Logrieco A.F. (2019). Mycotoxin risks under a climate change scenario in Europe. Trends Food Sci. Technol..

[40] Assunção R., Martins C., Viegas S., Viegas C., Jakobsen L.S., Pires S., Alvito P. (2018). Climate change and the health impact of aflatoxins exposure in Portugal—An overview. Food Addit. Contam. Part A.

[41] Sinha R.N., Jayas D.S., White N.D.G., Muir W.E. (1995). Stored grain ecosystem. Thestored-Grain Ecosystem.

[42] Magan N., Aldred D., Sanchis V., Arora D. (2003). Role of spoilage fungi in seed deterioration. Fungal Biotechnology in Agricultural, Food, and Environmental Applications.

[43] Venkateswarlu B., Shanker A.K. (2009). Climate change and agriculture: Adaptation and mitigation stategies. Indian J. Agron..

[44] Vuković A.J., Vujadinović M.P., Rendulić S.M., Đurđević V.S., Ruml M.M., Babić V.P., Popović D.P. (2018). Global warming impact on climate change in Serbia for the period 1961–2100. Therm. Sci..

[45] Petrović G., Karabašević D., Vukotić S., Mirčetić V., Radosavac A. (2020). The impact of climate change on the corn yield in Serbia. Acta Agric. Serb..

[46] Marinović I., Cindrić Kalin K., Güttler I., Pasarić Z. (2021). Dry spells in Croatia: Observed climate change and climate projections. Atmosphere.

[47] Miklin L., Podolszki L., Gulam V., Markotić I. (2022). The Impact of Climate Changes on Slope Stability and Landslide Conditioning Factors: An Example from Kravarsko, Croatia. Remote Sens..

[48] Lević J., Gošić-Dondo S., Ivanović D., Stanković S., Krnjaja V., Bočarov-Stančić A., Stepanić A. (2013). An outbreak of *Aspergillus* species in response to environmental conditions in Serbia. Pestic. Phytomedicine.

[49] Kos J., Lević J., Đuragić O., Kokić B., Miladinović I. (2014). Occurrence and estimation of aflatoxin M1 exposure in milk in Serbia. Food Control.

[50] Torović L. (2015). Aflatoxin M1 in processed milk and infant formulae and corresponding exposure of adult population in Serbia in 2013–2014. Food Addit. Contam. Part B.

[51] Milićević D.R., Spirić D., Radičević T., Velebit B., Stefanović S., Milojević L., Janković S. (2017). A review of the current situation of aflatoxin M1 in cow’s milk in Serbia: Risk assessment and regulatory aspects. Food Addit. Contam. Part A.

[52] Maslac T. (2013). US Department of Agriculture Grain and Feed, Annual Report on Wheat, Corn, and Barley. http://www.thefarmsite.com/reports/contents/sgmar13.pdf.

[53] Miocinovic J., Keskic T., Miloradovic Z., Kos A., Tomasevic I., Pudja P. (2017). The aflatoxin M1 crisis in the Serbian dairy sector: The year after. Food Addit. Contam. Part B.

[54] Olesen J.E., Trnka M., Kersebaum K.C., Skjelvåg A.O., Seguin B., Peltonen-Sainio P., Rossi F., Kozyra J., Micale F. (2011). Impacts and adaptation of European crop production systems to climate change. Eur. J. Agron..

[55] Bryden W.L. (2012). Food and feed, mycotoxins and the perpetual pentagram in a changing animal production environment. Anim. Prod. Sci..

[56] Santin E., Diaz D.E. (2005). Mould growth and mycotoxin production. Mycotoxin Blue Book.

[57] Bilandžić N., Božić Đ., Đokić M., Sedak M., Kolanović B.S., Varenina I., Cvetnić Ž. (2014). Assessment of aflatoxin M1 contamination in the milk of four dairy species in Croatia. Food Control.

[58] Bilandžić N., Varga I., Varenina I., Kolanović B.S., Božić Luburić Đ., Đokić M., Sedak M., Cvetnić Ž. (2022). Seasonal Occurrence of Aflatoxin M1 in Raw Milk during a Five-Year Period in Croatia: Dietary Exposure and Risk Assessment. Foods.

[59] Indexmundi. https://www.indexmundi.com/agriculture/.

[60] CEIC-Micro and Macroeconomic Data. https://www.ceicdata.com/en/croatia/agricultural-production-yield/agricultural-production-yield-late-crops-maize.

[61] Battilani P., Toscano P., der Fels-Klerx V., Moretti A., Camardo Leggieri M., Brera C., Rortais A., Goumperis T., Robinson T. (2016). Aflatoxin B1 contamination in maize in Europe increases due to climate change. Sci. Rep..

[62] Kresovic B., Matovic G., Gregoric E., Djuricin S., Bodroža D. (2014). Irrigation as a climate change impact mitigation measure: An agronomic and economic assessment of maize production in Serbia. Agric. Water Manag..

[63] Maslac T., US Department of Agriculture (USDA) Grain and Feed Annual (2021). Annual Report on Wheat, Corn and Barley for Serbia.

[64] Kovacevic V., Sostaric J. (2016). Impact of weather on the spring crops yield in Croatia with emphasis on climatic change and the 2014 growing season. Acta Agrar. Debr..

[65] Pandžić K., Likso T., Pejić I., Šarčević H., Pecina M., Šestak I., Tomšić D., Strelec Mahović N. (2022). Application of the self-calibrated palmer drought severity index and standardized precipitation index for estimation of drought impact on maize grain yield in Pannonian part of Croatia. Nat. Hazards.

[66] Grillakis M.G. (2019). Increase in severe and extreme soil moisture droughts for Europe under climate change. Sci. Total Environ..

[67] Lhotka O., Kyselý J., Farda A. (2018). Climate change scenarios of heat waves in Central Europe and their uncertainties. Theor. Appl. Climatol..

[68] Copernicus Climate Change Servise European State of The Climate. https://climate.copernicus.eu/ESOTC.

[69] The World Meteorological Organization. https://public.wmo.int/en/about-us.

